# Applying terror management theory to patients with life-threatening illness: a systematic review

**DOI:** 10.1186/s12904-023-01193-6

**Published:** 2023-06-17

**Authors:** Mark Svet, Laura B. Portalupi, Tom Pyszczynski, Daniel D. Matlock, Larry A. Allen

**Affiliations:** 1 School of Medicine, University of Colorado, Academic Office 1, Room 7019 12631 E. 17th Avenue, Mail Stop B130, Aurora, CO 80045 USA; 2 University of Colorado, Colorado Springs, CO USA

**Keywords:** Life-threatening illness, Terror management theory, Behavioral medicine, End-of-life care, Death anxiety

## Abstract

**Background:**

Terror management theory (TMT) posits that people manage death-related anxiety through the meaning provided by their cultural world-views and the sense of personal value provided by self-esteem. While a large body of research has supported the core propositions of TMT, little research has focused on its application to individuals with terminal illness. If TMT can help healthcare providers better understand how belief systems adapt and change in life-threatening illness, and the role they play in managing death-related anxiety, it may provide guidance on how to improve communication around treatments near the end of life. As such, we set out to review the available research articles that focus on describing the relationship between TMT and life-threatening illness.

**Methods:**

We reviewed PubMed, PsycINFO, Google Scholar, and EMBASE through May 2022 for original research articles focused on TMT and life-threatening illness. Articles were only deemed appropriate for inclusion if direct incorporation of the principles of TMT were made in reference to a population of interest whom had life-threatening illness Results were screened by title and abstract, followed by full review of candidate articles. References were also scanned. Articles were assessed qualitatively.

**Results:**

Six relevant and original research articles were published which provide varied levels of support for TMT’s application in critical illness, each article detailed evidence of ideological changes consistent with what TMT would predict. Building self-esteem, enhancing the experience of life as meaningful, incorporating spirituality, engaging family members, and caring for patients at home where meaning and self-esteem can be better maintained are strategies supported by the studies and serve as starting points for further research.

**Conclusion:**

These articles suggest that applying TMT to life-threatening illness can help identify psychological changes that may effectively minimize the distress from dying. Limitations of this study include a heterogenous group of relevant studies and qualitative assessment.

## Background

Terror management theory (TMT) was introduced in 1986 to explain how people cope with the knowledge that they will one day die. It posits that people manage the anxiety that is caused by the knowledge of the inevitability of death through the meaning provided by their cultural world-views and the self-esteem they acquire by living up to the standards of their worldviews. Research has shown that when individuals are confronted with their own mortality, they deepen their adherence to previously established world-views and personal beliefs as well as work harder to maintain self-esteem and close relationships in an effort to give meaning to one’s life. This adaptation reflects an effort to manage the conflict between an inherent desire for survival and the awareness of the impermanence of life, wherein deepening an allegiance to a system of meaning minimizes the psychological impact of impending death [1]. While more than 500 studies have applied this existential psychological theory to human behavior, most involve healthy individuals in experimental situations. Thus there is a relative lack of data assessing the applicability of TMT in individuals imminently near death.

This gap in understanding of how TMT might apply to people predictably near death is important given evidence that the care patients receive at the end of life may not be concordant with their wishes [2]. This discrepancy has been explored in the literature previously, and the Transtheoretical Model of Irrational Biomedical Exuberance (TRIBE) model emerged to postulate that standard recommendations from physicians to pursue aggressive medical therapies may also be related to physicians own death related anxiety [3, 4]. Additionally, end-of-life conversations are difficult and physicians often feel they lack an effective framework to have these discussions [5]. Together, these may explain the historic discrepancy between patient desires and the care they receive. In one study of patients with chronic kidney disease, 61% regretted initiating dialysis [6]. Another study found that in one chart review there was a discrepancy in desired code status (the desire to be resuscitated with CPR or not) in 22.7% of charts reviewed [7]. If TMT can help healthcare providers better understand how belief systems adapt and change in life-threatening illness, and the role they play in managing death-related anxiety, it may provide guidance on how to improve communication around treatments near the end of life. This becomes especially important as not all individuals are equipped with the existential maturity to navigate death’s salience on their own, and even prior attempts to create a uniform method to utilize palliative interventions across healthcare systems have failed with at least some contribution from a lack of evidentiary basis [8, 9]. Here we provide a systematic review of the studies that do exist in using TMT to shed light on responses to life-threatening illness to establish a foundational base for this area of investigation.

## Methods

PubMed, PsycINFO, Google Scholar, and EMBASE were searched through May 2022 for publications in which TMT was applied to life-threatening illness. No prespecified protocol was used. Search terms included ‘terror management theory’, ‘illness’, ‘disease’, ‘death’, ‘decision making’, and ‘end-of-life’. Results were screened by title and abstract, followed by full review of candidate articles. References were also scanned. This produced 17 relevant articles. We excluded five articles with insubstantial content, four essays, and two redundant secondary analyses. Articles were only deemed appropriate for inclusion if direct incorporation of the principles of TMT were made in reference to a population of interest whom had life-threatening illness as determined by individual, independent researchers. This left six original research reports which had their outcomes qualitatively assessed for this unregistered review (Table 1).

**Table 1:** Original research examining terror management theory (TMT) and life-threatening illness

Study/Location	Design	Main aim	Sample	Findings
Little and Sayers (2004) Australia	Qualitative analysis of interviews and published narratives	To define categories of experience related to death awareness in cancer survivors and their caregivers	17 cancer survivors; 3 partners and caregivers of cancer survivors	People who were mortality salient (aware of inevitability of death as shared eventual fate) turned outward for validation. People who were death salient (aware of inevitability of personal death) turned inward.
Edmondson, Park, Chaudoir, and Wortmann (2008) USA	Cross-sectional	To explore the validity of TMT in the context of chronic, intense mortality salience due to terminal illness	98 patients with end-stage^a^ congestive heart failure and some level of religiosity	Religious world views that provide meaning and value buffered death concerns in terminally ill people; a breakdown in religious world views increased vulnerability to terror of death.
Fernandez-Campos (2013) India^b^	Cross-sectional	To explore whether chronic exposure to death leads to death acceptance	30 patients with advanced terminal cancer and 29 farmers with no major health concerns	Both groups were more defensive of their world views after being asked to think about their own death, suggesting that having a terminal illness does not lead to death acceptance.
Neel, Lo, Rydall, Hales, and Rodin (2013) Canada	Cross-sectional	To measure death anxiety and determine the psychosocial and disease-related factors associated with it	60 outpatients with advanced cancer	Self-esteem served as a protective factor against death anxiety in people for whom death was imminent.
Willis, Mah, Shapiro, Hales, Li, An, Zimmermann, Schutlebraucks, Rodin (2021) USA	Secondary analysis of a longitudinal RCT	To assess the effect of TMT defense mechanisms on death anxiety in those with advanced cancer	305 patients with advanced cancer	The defense mechanisms described by TMT buffer against the death anxiety experienced by those with terminal conditions
Hong, Yuhan, Youhui, Zhanyin, Shili, Xiaoting, Wenhua (2022) China	Cross-sectional	To explore the relationship between factors predicted by TMT to be defense mechanisms and the relationship to death anxiety	270 patients with advanced cancer	Patients with high self-esteem, resilience, and adult children reported lower death anxiety, suggesting a possible protective factor

^a^The authors defined end-stage as heart failure patients with New York Heart Association class III or IV.

^b^This dissertation reports the most comprehensive and recent results of this research, comprising two experiments. Published results of the first experiment can be found here: Fernandez, S., Castano, E., & Singh, I. (2010). Managing death in the burning grounds of Varanasi, India: A terror management investigation. *Journal of Cross-Cultural Psychology*, *41*, 182–194. 10.1177/0022022109354376

## Results

The studies found varied levels of support for TMT in life-threatening illness. Edmondson et al. performed a cross-sectional study exploring the validity of TMT in 98 people with end-stage heart failure by distributing standardized questionnaires that assessed levels of depressive symptoms, concerns surrounding death, and spirituality. They showed that properly functioning religious beliefs, an especially important aspect of many people’s world-views, buffered against death concerns and ultimately depression due to their role as a terror management mechanism [10]. Neel et al. performed a cross-sectional study of 60 people with metastatic cancer, also using standardized questionnaires, to gauge levels of death anxiety as well as self-esteem; they found that self-esteem served as a protective factor against death anxiety consistent with TMT [11]. Fernandez-Campos published a study conducted in Varanasi, considered the spiritual capital of India, that studied the effect of a reminder of death in 30 people with terminal cancer and 29 people with no major health concerns and found elevated levels of world-view defense (measured via attachment to India on a standardized scale) after this prompt [12]. Notably, this adaptation was present in both terminally ill patients and healthy patients, suggesting these psychological changes do not wane with chronic exposure to illness. Little et al. performed extended narrative interviews with cancer patients and found both they and their caregivers undergoing active treatment tended to turn to their close friends and family who have shared values—as TMT would predict [13]. Willis et al. published longitudinal research around 305 patients with advanced cancer showing that individuals with several of the defense mechanisms described by TMT, specifically attachment security, meaning in life, and self-esteem, had lower rates of physical impairment and distress associated with dying [14]. Finally, Hong et al. published a cross sectional study using standardized surveys focusing on factors predicted by TMT to be anxiety buffering, such as levels of self-esteem, resilience, and familial support, among 270 patients with advanced cancer and found these inversely correlated with the degree of anxiety surrounding their death [15].

## Discussion

At its core, TMT posits the idea that confrontation with one’s own mortality poses an existential crisis that sets in motion behavioral and psychological changes to minimize distress associated with dying. Research has shown that finding meaning in one’s cultural worldview, self-esteem by adhering to the values of one’s worldview, and the comfort and validation provided by close interpersonal relationships provides comfort in the face of knowledge of one’s mortality. While the vast majority of research testing this theory comes from among healthy individuals, this systematic review provides evidence from the few extant studies that have assessed the applicability of these ideas to people with life-threatening illness. These studies suggest that similar processes and protective mechanisms play out among such people. Because the results of these studies show the potential utility of TMT for working with terminally ill, additional research in this domain is sorely needed.

To further emphasize this point, as recently described by Perry et al., there is a barrier to delivering comprehensive emotional and physical support to patients who are terminally ill. The death related anxiety experienced by patients and described by TMT tends to dissuade both patients and physicians from engaging in these conversations. The fact that only 20–30% of patients engage in advanced care planning is supportive of this. This can be interpreted in the lens of TMT as a maladaptive response to the inevitability of death [16]. However, the emotional mechanisms underlying this behavior can also be capitalized upon to better deliver end of life care through the utilization of the principles of TMT. In fact, significant research exists on this topic specifically surrounding Dignity Therapy and Meaning Centered Psychotherapy which focuses on interventions to foster a sense of self-esteem and self-worth in the face of life-threatening illnesses [17].

The studies we describe, introduce the possibility of applying TMT to life-threatening illness to help predict behavioral responses and guide treatment strategies for terminally ill individuals. In this way, TMT can provide a framework for identifying therapeutic targets to decrease death anxiety and thereby improve quality of life. Interventions that would specifically address these adaptations such as building self-esteem, helping patients find greater meaning in both their past life and their journey through their illness, involving family members at clinic visits and in discussions of treatment plans, and focusing on less invasive treatment strategies while emphasizing comfort and care at home.

For many, but not all patients, incorporating religious support such as chaplains in treatment plans may be an especially useful way of achieving these goals. As it currently stands, few components of these described psychological changes are regularly targeted and chaplains are currently only utilized ~ 52% of the time in goals-of-care conversations [18]. While further studies would be needed to confirm the efficacy of such interventions (such as those centered around dignity therapy 19), there already exists some utility behind such an approach evidenced through the Life Tape Project (LTP). This project used tape recordings of clinic visits wherein cancer diagnoses are explained for the first time and families are given the opportunity to recount their life-story as a means of strengthening family bonds and support. This intervention was found to improve quality of life among these patients which was attributed at least in part to fostering a form of symbolic immortality, a result that is consistent with the application of TMT and holds promise for its further use [20]. This sort of a holistic approach may also help explain, at least in part, the effectiveness of palliative care interventions that facilitate social connection and identify meaningful ways to contribute within one’s social group. Combined with prior research showing physician communication is a trainable skill [21], incorporating TMT within existing frameworks to develop a better understanding of adaptive behavioural and psychological changes among those with life-threatening illness may ultimately lead to improved quality of life. While further research is needed, this better understanding may also lead to more reliable goals-of-care conversations, thereby limiting discrepancies between desired and delivered care.

One of the main limitations of this review is in the heterogeneity of the included studies and variable study protocols of the incorporated research. While this likely stems from the paucity of relevant research articles; this limits the assessment of bias and sensitivity of these studies to systematic review. This further emphasizes the importance of ongoing research in this area, particularly in a standardized way.

## Conclusions

While a large body of research has provided evidence supporting the core propositions of TMT, little research has previously focused on its application to individuals with terminal illness. Our research has provided a review of the research that is available, thereby adding to the body of literature supporting TMT as a mechanism to understand how people adapt their values to manage death-related anxiety. Using this theory to understand how belief systems adapt and change in life-threatening illness, may enable healthcare providers to utilize different strategies and approaches to better provide goal-concordant care.

## Data Availability

All data generated or analysed during this study is included in this published article. The raw, de-identified data may be made available upon reasonable request from the corresponding authors.

## List of abbreviations

CPR: Cardiopulmonary resuscitation
LTP: Life-Tape Project
TMT: Terror management theory
